# Identifying Longitudinal Trajectories of Quality of Life and Associated Risk and Protective Factors Among Cancer Patients

**DOI:** 10.1002/pon.70453

**Published:** 2026-04-06

**Authors:** Jiwon Kim, Karen Llave, Maja Kuharic, Nicola Lancki, Kathryn Jackson, Kimberly A. Webster, David Cella

**Affiliations:** ^1^ Department of Medical Social Sciences Northwestern University Feinberg School of Medicine Chicago Illinois USA; ^2^ Department of Population Health & Disease Prevention University of Calinfornia Irvine California USA; ^3^ Department of Preventive Medicine Northwestern University Feinberg School of Medicine Chicago Illinois USA

**Keywords:** cancer, FACT‐G7, longitudinal studies, oncology, protective factors, quality of life, risk factors

## Abstract

**Objective:**

The 7‐item Functional Assessment of Cancer Therapy‐General (FACT‐G7) is a validated, brief measure of health‐related quality of life (QoL) used in oncology settings. While many conceptualize QoL as a static trial endpoint, growing evidence underscores its dynamic nature over time to inform point‐of‐care interventions. Grounded in the Wilson and Cleary model, this study aims to (1) identify distinct trajectories of QoL over a 12‐month period using FACT‐G7, and (2) incorporate clinical, mental health, sociodemographic, and healthcare system related factors to uncover risk and protective factors that shape patients' QoL trajectories.

**Methods:**

Using FACT‐G7 scores over a 12‐month period from a sample of 4104 cancer patients (aged 19–92, *M* = 60.50, SD = 12.95) receiving cancer treatment, growth mixture modeling was fitted to identify subgroups of QoL trajectories. Multinomial logistic regression was conducted to examine the predictors of class membership.

**Results:**

Results revealed a three‐class model provided optimal fit: high QoL (40.1%), average QoL (40.3%), and low QoL (19.6%). Psychosocial factors ‐ particularly loneliness (OR = 1.70, 95% CI:1.35–2.13) and financial difficulties (OR = 1.47, 95% CI:1.27–1.71) ‐ strongly predicted membership in the low QoL trajectory. Clinical factors, including comorbidities (Charlson Comorbidity Index; CCI) and symptom burden severity (PRO‐CTCAE), were associated with poorer QoL trajectories, with insomnia and nausea demonstrating the strongest negative effects. Conversely, higher satisfaction with cancer care and cancer‐related self‐efficacy were protective factors associated with higher QoL trajectories.

**Conclusions:**

The findings identify modifiable risk and protective factors that can inform targeted early interventions to improve long‐term QoL outcomes across the cancer care continuum.

## 1 Background

Quality of life (QoL) is a multidimensional concept that reflects patients' subjective perceptions of their symptoms across physical, psychological and social domains [1, 2]. Patients often experience significant fluctuations in their QoL throughout cancer diagnosis, treatment, and survivorship [3, 4]. In oncology, QoL assessment has become increasingly recognized as a critical outcome measure alongside traditional clinical endpoints such as survival and tumor response. Patient‐reported QoL captures the effectiveness and burden of treatments, reflects patients' ability to function in daily life, and informs shared decision‐making by incorporating patients' personal values and perceptions of care [5]. In addition, patient‐reported QoL can also be used to predict other outcomes such as mortality, healthcare utilization, and treatment discontinuation [6, 7].

Wilson & Cleary's conceptual model of QoL demonstrates a causal pathway between biological and physiological variables, symptom status, functional status, general health perceptions, and overall QoL [8]. The biological and physiological factors refer to the functioning of cells, organs, and organ systems. Symptom status refers to the patient's perception of an abnormal state in physical, emotional, or cognitive domains. Functional status is defined as an individual's ability to perform specific tasks and is significantly influenced by symptom status. It includes physical, social, role, and psychological functioning, all of which are also highly influenced by the social environmental factors. General health perceptions are an integration of all previous health variables, including other subjective variables such as mental health. Finally, QoL, located at the end of the continuum, represents a patient's overall assessment of all factors.

Wilson and Cleary's model has been widely incorporated in cross‐sectional studies to identify meaningful factors influencing QoL across cancer populations [9]. Although QoL is frequently assessed at a single time point, it is inherently a dynamic construct that is expected to fluctuate throughout the cancer care continuum. Understanding its longitudinal nature is important for identifying at‐risk subgroups and informing timely, personalized interventions. Recent studies have started exploring the longitudinal patterns of QoL over time, revealing that patients do not follow a uniform pattern of decline or improvement in QoL [10, 11, 12, 13].

Trajectories of QoL have been examined in various cancer contexts, including breast cancer patients at diagnosis and after 1 year of treatment [10, 11], and for palliative care at the patient's last year of life [13]. In these studies, QoL was measured using the 27‐item Functional Assessment of Cancer Therapy—General (FACT‐G), a widely used cancer‐specific instrument for assessing physical, emotional, social, and functional well‐being [1]. The number of trajectories varied across the studies. For example, among breast cancer patients at the time of diagnosis, two trajectories of consistently low (41%) and medium (59%) QoL groups were identified. After 1 year of treatment, the study identified three trajectory groups: low (21%), medium (45%), and high (34%) [10, 11]. Finally, QoL patterns observed during patient's last year of life revealed four trajectories: overall high (47%), progressively decreasing (32%), asymmetric decline (13%), and overall low (8%) [13]. These findings illustrate the sensitivity of the cancer‐specific FACT‐G in capturing distinct and clinically meaningful QoL trajectory groups over time.

Other studies have employed different QoL measures. For instance, one study identified latent subgroups of metastatic colorectal cancer patients using longitudinal QoL data measured by the European Organization for Research and Treatment of Cancer Quality of Life Questionnaire‐Core 30 (EORTC‐QLQ‐C30), revealing three patterns: high‐stable, intermediate, and low‐declining [12]. Another study, though not explicitly identifying trajectory groups of QoL, used the European Quality of Life 5 Dimensions 5 Level Version (EQ‐5D‐5L) to assess QoL among cancer patients until death, alongside the EORTC‐QLQ‐C30 for up to 1 year [14]. Findings indicated that QoL remained relatively stable for most months, but deteriorated considerably in the last 3 months of life on both instruments.

Prior studies have often relied on relatively small sample sizes (100–300), which limit the generalizability and robustness of findings, particularly when applying complex analysis methods such as growth mixture models. Furthermore, most previous studies focused narrowly on specific cancer types or treatment stages, highlighting the need to explore the broader patterns of QoL across different cancer types and cancer care continuum. In the current study, we use longitudinal data collected from a large and diverse sample of cancer patients, allowing for a more reliable estimation of QoL trajectories over time. FACT‐G7 is employed as a brief, valid, and cancer‐specific measure of QoL that is particularly well‐suited for longitudinal research due to its sensitivity and responsiveness to change and low respondent burden [15]. Additionally, this study incorporates a wide range of predictors including clinical, psychosocial, sociodemographic, and healthcare system related variables to identify both risk and protective factors of the longitudinal QoL trajectories.

Therefore, the objectives of this study are (1) to identify distinct trajectories of QoL in cancer patients over a 12‐month period using the FACT‐G7; and (2) to examine whether key clinical, psychosocial, sociodemographic, and healthcare system related factors at baseline predict membership in these different QoL trajectories.

## 2 Methods

### 2.1 Study Design

This study employed data from the Northwestern University IMPACT (Improving the Management of Symptoms during and Following Cancer Treatment) [16] study; a stepped‐wedge cluster‐randomized trial with an embedded randomized controlled trial designed to evaluate implementation and effectiveness outcomes for an electronic health record (EHR) embedded symptom monitoring and management program for outpatient cancer care.

#### 2.1.1 Participants and Recruitment

As part of the IMPACT study [16], patients were prospectively recruited and those who consented were asked to complete comprehensive baseline and quarterly surveys, as well as monthly PRO assessments administered via REDCap over a 12 month period. Eligibility criteria included (1) age 18 years or older, (2) a cancer diagnosis within the last 10 years, (3) had a visit at a Northwestern clinic in the past year, (4) ability to read English or Spanish, and (5) a valid email address. Patients across the cancer care continuum were included in the study, because symptom burden and self‐management needs persist beyond active treatment. The study procedures were approved by the Northwestern University Institutional Review Board (STU00208413) and informed consent was obtained from all participants.

### 2.2 Data Collection

The outcome variable for examining the QoL trajectories was measured at five time points, ranging from baseline, 3 months, 6 months, 9 months, to 12 months. All predictors of group membership ‐ clinical, psychosocial, sociodemographic, and healthcare system related factors ‐ were from baseline. We chose to focus on the baseline predictors to identify early, clinically accessible factors associated with longitudinal QoL trajectories. Time‐varying covariates were not included due to substantial missingness at follow‐up and increased model complexity. Study data were collected and managed using REDCap hosted at Northwestern University [17, 18]. REDCap (Research Electronic Data Capture) is a secure, web‐based software platform designed to support data capture for research studies, providing (1) an intuitive interface for validated data capture; (2) audit trails for tracking data manipulation and export procedures; (3) automated exported procedures for seamless data downloads to common statistical packages; and (4) procedures for data integration and interoperability with external sources.

#### 2.2.1 Outcome Variable

The Functional Assessment of Cancer Therapy ‐ General ‐ 7 Item version (FACT‐G7) is a brief measure of health‐related quality of life designed for use in oncology settings with cancer patients aged 18 years and older [19]. FACT‐G7 is a shortened form of the original FACT‐G, one of the most frequently used measure of QoL in oncology settings [20]. It includes seven items with a 7‐day recall period, including lack of energy, pain, nausea, worry about worsening condition, sleep quality, enjoyment of life, and contentment with QoL. Responses range from 0 = Not at all to 4 = Very much, yielding a total score of 0–28, where higher scores indicate better QoL. The FACT‐G7 is an excellent predictor of the longer, widely‐used 27‐item FACT‐G [19].

#### 2.2.2 Clinical Factors

The Charlson Comorbidity Index (CCI) was utilized as a measure of mortality risk and burden of disease. The score is calculated based on the weighted points of 17 conditions and additional weighted points assigned for age [21, 22]. The conditions were identified in the patients' electronic health record from ICD‐9 and ICD‐10 codes at the time of enrollment. The scores in the current study ranged from 0 to 19, with higher scores indicating greater risk of mortality. For the Patient Reported Outcomes version of the Common Terminology Criteria for Adverse Events (PRO‐CTCAE), participants were asked about the frequency and severity of specific symptoms experienced in the past 7 days [23]. Specifically, the frequency of nausea was rated with response options ranging from 0 = Never to 4 = Almost Constantly, and severity of constipation, shortness of breath, and insomnia at its worst was rated as individual items with response options ranging from 0 = None to 4 = Very Severe.

#### 2.2.3 Psychosocial Factors

For loneliness, a single item from the UCLA loneliness scale (“I feel isolated from others”) was used, with the scale ranging from 1 = Never to 5 = Always [24]. For depression and anxiety, PROMIS Depression and Anxiety item banks (v1.0) were administered using Computerized Adaptive Testing (CAT), and T‐scores with a mean of 50 and standard deviation of 10 were used. For self‐efficacy, the Communication and Attitudinal Self‐Efficacy (CASE) scale for cancer was employed, consisting of four items: “If I don't understand something, it is easy for me to ask for help”, “It is easy for me to ask nurses questions”, “It is easy for me to ask my doctor questions”, and “It is easy for me to get information about cancer” [25]. Response options ranged from 1 = Strongly disagree to 4 = Strongly agree, and the total sum score was used.

#### 2.2.4 Sociodemographic Factors

Sociodemographic variables were collected from the EHR: age and sex; or by self‐report in the baseline survey: education (0 = high school or less, 1 = some college or higher), employment status (0 = Not working, including retired, homemaker, on disability or leave of absence, unemployed, or full‐time student, 1 = Full/part time), and health insurance (Medicaid and Medicare vs. all other types). Financial difficulty was measured using a single item on Financial Toxicity (“My illness has been a financial hardship to my family and me”; 0 = Not at all to 4 = Very Much).

#### 2.2.5 Healthcare System Factors

Shared decision making was measured using the CollaboRATE scale [26]. Participants rated how much effort was made by their care team to help them understand their health issues, listen to what matters most to them, and include those priorities in next‐step decisions over the past 3 months. Responses ranged from 0 = No effort to 4 = Every effort, and scores were dichotomized using a “Top Box” approach (1 = all items scored 4; 0 = otherwise). The overall cancer care experience was assessed using the Consumer Assessment of Healthcare Providers and Systems (CAHPS 42) item for cancer care [27]. Responses ranged from 0 to 10, with higher scores indicating more positive perceptions of care. Participants were also asked to report on their healthcare utilization over the past 6 months (0 = No, 1 = Yes). Treatment intent was identified using data from the Beacon treatment plan that was active at the time of recruitment from the electronic health record (EPIC), and was categorized as curative or non‐curative.

### 2.3 Conceptual Model

This study is guided by the conceptual model framed by Wilson and Cleary [8], which outlines a causal pathway linking biological/physiological factors, symptom status, functional status, and general health perceptions to the overall QoL [8]. This framework posits that QoL is not directly determined by disease characteristics alone, but rather mediated through patient's symptom experiences, functional abilities, and subjective health perceptions. The model also acknowledges that individual characteristics and environment factors can modify relationships within the causal pathway.

**FIGURE 1:**
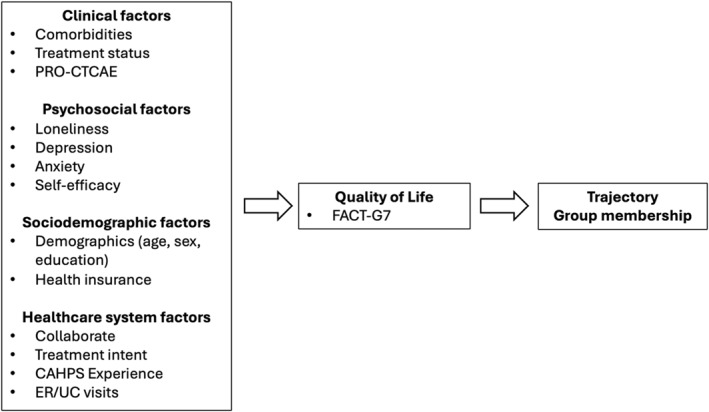
Conceptual model.

We extend the Wilson and Cleary framework in two important ways relevant to QoL in the cancer care continuum, as depicted in Figure 1. First, we expand the model's content scope by incorporating a broader range of predictors of QoL including clinical, psychosocial, sociodemographic, and healthcare‐related factors. In particular, we incorporate healthcare‐related and sociodemographic factors, such as social determinants of health, healthcare utilization, and care processes, which were not systematically operationalized in the original framework, allowing for a more comprehensive representation of contextual influences on QoL in cancer care. Second, we extend the temporal scope of the framework by focusing on the longitudinal QoL trajectory patterns as the outcome rather than QoL measured at a single time point. Longitudinal assessments of FACT‐G7 collected over 12 months were used to identify distinct patterns of change. Our extended model leverages the conceptual framework of the Wilson and Cleary model to uncover meaningful QoL trajectory patterns and their associated baseline characteristics.

#### 2.3.1 Predictor Selection and Hypothesized Directions

The selection of predictors was guided by the Wilson and Cleary model to ensure comprehensive coverage of factors across the causal pathway, as well as individual and environmental contextual characteristics that modify the pathway. First, clinical factors were selected to represent the biological/physiological and symptom domains of the model. Greater comorbidity burden (CCI) was hypothesized to predict lower or declining QoL trajectories, as multiple chronic conditions amplify symptom severity and functional decline. Greater frequency or severity of PRO‐CTCAE was expected to predict lower QoL trajectories, as they directly constrain functional status and lead to diminished overall QoL.

Second, psychosocial factors were selected to reflect the psychological and social symptoms that indirectly influence functional status, general health perceptions, and overall QoL. Higher levels of depression and anxiety were hypothesized to predict poorer QoL trajectories as indicators of affective symptom burden. Greater loneliness was expected to predict lower QoL trajectories, reflecting difficulties in social functioning and support. Conversely, higher self‐efficacy (CASE) scores were hypothesized to predict more favorable trajectories through enhanced care engagement and proactive symptom management.

Third, sociodemographic and healthcare system factors were selected to represent the contextual characteristics and environmental factors within the Wilson and Cleary framework. Regarding sociodemographic factors, higher education was hypothesized to predict better QoL trajectories through greater health literacy, whereas greater financial toxicity was expected to predict poorer trajectories due to increased stress and barriers to treatment adherence. Employed status was expected to predict favorable trajectories, reflecting greater functional capacity and financial stability, whereas Medicare and Medicaid coverage were expected to predict less favorable trajectories due to socioeconomic vulnerabilities. Age and sex were included to account for potential differences in symptom experience, without a specific directional hypothesis.

Regarding healthcare system factors, positive experiences in shared decision making (CollaboRATE) and overall cancer care (CAHPS) were expected to predict better trajectories by enhancing symptom management and improving patient perception. Curative treatment intent was expected to predict better trajectories, reflecting better prognosis and likelihood of symptom resolution. On the other hand, healthcare utilizations, including ER visits and inpatient stays, were hypothesized to predict poorer trajectories as indicators of acute care needs and disease‐related complications.

Overall, the conceptual model in Figure 1 hypothesizes that trajectory group membership is influenced by the interplay of the four domains. While we use the Wilson and Cleary framework as a conceptual guide for organizing the structure for predictor selections, our analytic strategy focuses on prognostic prediction through simultaneous inclusion of all predictors, rather than testing the causal pathways specified by the framework. By identifying distinct trajectory groups and their associated baseline characteristics, our extended model and analytic approach aims to identify clinically actionable factors associated with unfavorable QoL trajectories to support targeted supportive care and early risk stratification.

### 2.4 Analytic Approach

Growth Mixture Modeling (GMM) is a person‐centered approach that identifies latent classes or subgroups of individuals who show similar patterns of change over time [28]. Unlike conventional longitudinal modeling approaches that assume a homogeneous population trajectory, GMM uncovers subgroups within the overall sample that exhibit distinct growth trajectories. This classification offers meaningful insights for tailoring interventions to meet the specific needs of different subpopulations. We employ a three‐step approach, a recommended method for examining auxiliary variables in mixture models while preserving the latent class structure [29]. In the first step, the optimal number of latent classes is identified from unconditional GMM estimated using only the indicators. In the second step, individuals are assigned to their most likely class membership based on the full posterior probability distribution, and the associated classification errors are computed. In the third step, the most likely class variable is used for a secondary analysis (multinomial logistic regression), taking into account measurement uncertainty and avoiding the shift of latent class formation due to a simultaneous, joint modeling of all indicators and covariates.

To address the first aim of identifying distinct QoL trajectories, we estimated a series of unconditional latent class growth models and selected the optimal number of latent classes based on fit indices, likelihood ratio test, classification quality, and interpretability. Model fit was assessed using the Akaike Information Criterion (AIC) [30], Bayesian Information Criterion (BIC) [31], and sample‐size adjusted BIC [32], with lower values indicating better fit. For model comparison, the Lo‐Mendell‐Rubin Likelihood Ratio Tests (LMR LRT) and Bootstrap Likelihood Ratio Tests (BLRT) were used to evaluate whether a model with *k* classes provided a significantly better fit than a model with *k‐1* classes [33, 34]. A significant *p*‐value (< 0.05) suggests that the model with *k* classes provides a better fit. Classification quality was assessed with Entropy, with higher values indicating better classification.

To address the second aim of examining the effect of key factors of the QoL trajectories, all predictors were simultaneously entered into a multinomial logistic regression using a three‐step approach. After determining the optimal number of latent classes in the unconditional model, the most likely latent class membership was used as a dependent variable in a multinomial logistic regression, with all baseline clinical, psychosocial, sociodemographic, and healthcare system related factors entered simultaneously as predictors. Domains were not modeled as latent constructs nor added sequentially.

Missing data in the unconditional growth mixture model were handled using full‐information maximum likelihood (FIML) in Mplus, which incorporates all available data from participants who provided at least one FACT‐G7 assessment (*N* = 3819). For subsequent multinomial logistic regression, Mplus R3STEP procedure was employed, which applies listwise deletion and requires complete data on all covariates (*N* = 3113). Data cleaning and analysis were conducted using the statistical software R and Mplus [35, 36].

## 3 Results

### 3.1 Participant Characteristics

The baseline descriptive statistics are presented in Table 1 (*N* = 4104). Participants ranged in ages from 19 to 92 years (Mean = 60.50, SD = 12.95), with the majority of the sample being female (66%), non‐Hispanic/Latino (84%), and White (84%). Over 62% of the sample had attained a college degree or higher, and approximately 41% were employed either full‐time or part‐time. Regarding health insurance coverage, 49% had private insurance, 37% were covered by Medicare, and 3% were enrolled in Medicaid.

**TABLE 1:** Baseline descriptives (*N* = 4104).

Variables	Range	Mean (SD)	*N*	**%**
Socio‐demographics				
Age	19–92	60.50 (12.95)		
Sex				
Female			2719	66%
Male			1385	34%
Ethnicity				
Hispanic/Latino			174	4%
Not Hispanic/Latino			3461	84%
Unknown/Not reported			470	11%
Race				
White			3438	84%
Black/African American			218	5%
Other			124	3%
Unknown/Not reported			323	8%
Education				
High school or less			266	7%
Some college/Technical/Associate degree			886	22%
College graduate			1304	32%
Graduate/Advanced degree			1250	30%
Missing/Not reported			398	10%
Employment				
Full‐time employed			1305	32%
Part‐time employed			359	9%
Retired			1381	34%
Homemaker			159	4%
On disability or on leave of absence			337	8%
Unemployed or full‐time student			149	4%
Prefer not to answer/Not reported			379	9%
Health insurance				
Medicaid			127	3%
Medicare			1497	37%
Private			1997	49%
Other^a^			49	1%
Don't know/Not reported			434	11%
Financial difficulties	1–5	1.97 (1.21)		
Clinical factors				
Charlson comorbidity index (CCI)	0–19	5.94 (3.03)		
PRO‐CTCAE				
Nausea (frequency)	0–4	0.27 (0.70)		
Constipation (severity)	0–4	0.56 (0.87)		
Shortness of breath (severity)	0–4	0.55 (0.80)		
Insomnia (severity)	0–4	1.11 (1.01)		
Mental health				
Loneliness	1–5	1.98 (0.97)		
Depression T‐score	34.2–81.7	49.04 (8.41)		
Anxiety T‐score	32.9–84.9	51.24 (9.25)		
Self‐efficacy (CASE^b^)	4–16	14.95 (2.27)		
Healthcare system				
CollaboRATE				
Less than top score			2331	57%
Top score to all three items			1200	29%
Missing/Not reported			573	14%
Treatment intent				
Curative			800	19%
Non‐curative			474	12%
No beacon Tx plan			2830	69%
Hospital stays			1857	52%
ER visits			1010	28%
CAHPS experience	0–10	9.00 (1.51)		

^a^ Uninsured, self‐pay, military, or veteran sponsored.

^b^ Communication and Attitudinal Self‐Efficacy scale for cancer.

### 3.2 Missing Data

Table 2 presents the missing data rate for FACT‐G7 across the five time points. Of the 4104 participants enrolled at baseline, missing rates increased over time, with 12.6% at baseline, 28.1% at 3 months, 37.0% at 6 months, 41.4% at 9 months, and 42.9% at 12 months. This attrition rate is consistent with prior longitudinal oncology studies, including supportive care and palliative oncology trials, where around half of the participants drop out before the end of the study [37].

**TABLE 2:** Missing data rates for FACT‐G7 (*N* = 4104).

	Available *N*	Missing	**%**
Baseline	3586	518	12.6%
3 months	2950	1154	28.1%
6 months	2584	1520	37.0%
9 months	2406	1698	41.4%
12 months	2343	1761	42.9%

Overall, 1704 (42%) had complete FACT‐G7 data across all assessment waves. Among the 2400 participants with missing data, the missing pattern was mostly monotone (*n* = 1,183, 49%). 285 participants (12%) were missing at all time points, and 932 participants exhibited non‐monotone missingness (39%). The predominance of monotone missingness indicates that attrition was the primary source of missing data and represents a potential source of bias if dropout was related to unobserved outcomes.

The unconditional growth mixture models were fitted using FIML, which utilizes all available data from participants who provided at least one FACT‐G7 assessment (*N* = 3819). To evaluate the robustness of the trajectory results to missing data and attrition, we conducted a complete‐case sensitivity analysis by re‐estimating the latent classes using only participants with complete FACT‐G7 data across all given time points (*n* = 1704). Trajectory patterns, growth parameter estimates, and class proportions were highly consistent across the two results, supporting the robustness of the identified trajectory structure and the appropriateness of FIML for handling missing data in the present study.

### 3.3 Model Selection

A comparison of model fit indices, likelihood ratio tests, and entropy values for models with two to five classes is presented in Table 3. The model fit indices of AIC, BIC, and SABIC showed a decreasing trend as the number of latent classes increased. Notably, the largest decrease was observed between 2‐class and 3‐class models, with substantially smaller decrements thereafter. All likelihood ratio tests were significant, except LMR‐LRT for the comparison between the 5‐class and 4‐class models. Entropy values, indicating classification quality, were high (0.8 or above) for both 2‐class and 3‐class models, but dropped below 0.8 for the 4‐class and 5‐class models. Further examination of the class proportions revealed that the smallest class in the 4‐class and 5‐class models comprised approximately 8% and 5% of the participants, respectively, raising concerns about class over‐extraction and model stability.

**TABLE 3:** Comparison of two‐to five‐class models.

Criteria	2‐class	3‐class	4‐class	5‐class
Model fit indices				
AIC	78,292.734	75,879.377	74,797.491	74,458.303
BIC	78,355.212	75,960.598	74,897.455	74,577.010
SABIC	78,323.436	75,919.290	74,846.615	74,516.637
Likelihood ratio test				
LMR‐LRT	0.00	0.00	0.00	0.20
BLRT	0.00	0.00	0.00	0.00
Entropy	0.83	0.80	0.78	0.74

Based on the results of the model fit indices and clinical interpretability, the 3‐class model provided the most meaningful differentiation of the QoL trajectories with proportional class sizes and was selected as the final, optimal model. The final model was estimated with 500 random starts and 100 final‐stage optimizations, and the best log‐likelihood was replicated, indicating convergence to a stable solution. Average posterior probabilities for most likely class membership were high for all classes (0.87–0.93), while the cross‐classification probabilities were low.

### 3.4 Quality of Life Trajectories

**FIGURE 2:**
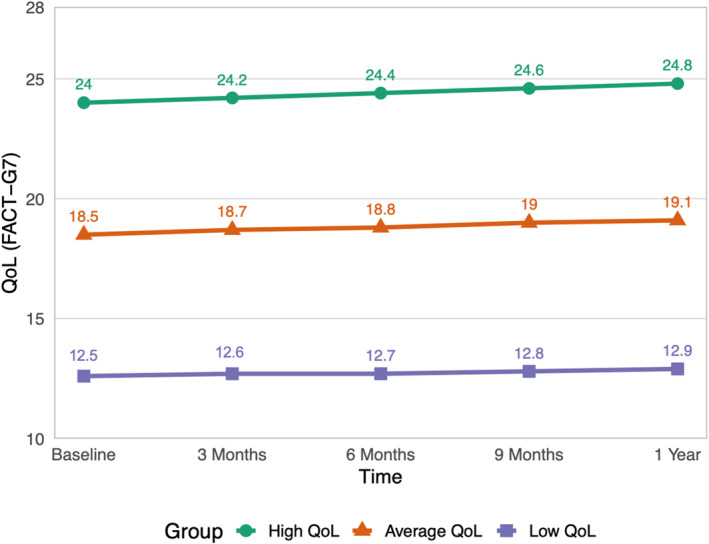
Growth trajectories of quality of life (FACT‐G7).

The three latent classes were characterized as: low, average, and high QoL groups. The estimated trajectories of each latent class are illustrated in Figure 2. The green solid line with circles at the top represents the high QoL group, comprising 40.1% of the participants. This group had an estimated FACT‐G7 score of 24 at baseline, which increased to 24.8 at 12 months. The orange solid line with triangles denotes the average QoL group, accounting for 40.3% of the sample. Their estimated FACT‐G7 score at baseline was 18.5, which gradually increased to 19.1 in 12 months. Lastly, the purple solid line with rectangles represents the low QoL group, comprising 19.6% of the sample. This smallest group had the lowest estimated baseline score of 12.5, with only a slight increase to 12.9 in 12 months, marking the smallest improvement among the three groups.

### 3.5 Predictors of Trajectory Membership

A multinomial logistic regression was conducted using group membership (low, average, high) as the dependent variable. The average QoL group was used as the reference group. The final sample size used for the multinomial logistic regression was 3,113, due to missing data on predictor variables. Tables 4 and 5 present the odds ratios (OR) with their standard error (SE), 95% confidence intervals, and the *p*‐values for each of the predictors.

**TABLE 4:** Predictors of trajectory groups (high QoL vs. average QoL, *N* = 3113).

High QoL (ref: Average QoL)					
Predictor	Odds ratio	SE	Upper CI	Lower CI	*p*‐value
Age	1.01	0.01	1.03	1.00	0.12
Female	1.23	0.15	1.64	0.91	0.17
College	1.89	0.26	3.17	1.13	0.02
Employed	1.09	0.18	1.54	0.78	0.61
Medicaid	0.66	0.40	1.45	0.30	0.31
Medicare	0.83	0.21	1.25	0.56	0.38
Curative	0.74	0.18	1.04	0.52	0.08
Non‐curative	0.75	0.25	1.23	0.45	0.25
CollaboRATE	1.38	0.15	1.86	1.02	0.04
ER visits	0.69	0.14	0.91	0.52	0.01
Hospital stays	0.44	0.17	0.62	0.32	0.00
CAHPS42	1.24	0.06	1.40	1.09	0.00
Financial difficulty	0.66	0.08	0.77	0.56	0.00
Loneliness	0.62	0.10	0.75	0.51	0.00
Depression	0.90	0.02	0.93	0.87	0.00
Anxiety	0.96	0.01	0.99	0.94	0.01
Self‐efficacy	1.00	0.03	1.06	0.94	0.95
Nausea	0.55	0.20	0.81	0.37	0.00
Constipation	0.77	0.10	0.94	0.64	0.01
Shortness of breath	0.55	0.11	0.68	0.44	0.00
Insomnia	0.41	0.09	0.49	0.35	0.00
CCI^a^	0.93	0.03	0.99	0.87	0.02

^a^ Charlson Comorbidity Index.

**TABLE 5:** Predictors of trajectory groups (low QoL vs. average QoL, *N* = 3113).

Low QoL (ref: Average QoL)					
Predictor	Odds ratio	SE	Upper CI	Lower CI	*p*‐value
Age	1.01	0.01	1.03	0.98	0.61
Female	1.46	0.20	2.16	0.99	0.06
College	0.93	0.33	1.76	0.49	0.82
Employed	1.05	0.22	1.62	0.69	0.81
Medicaid	2.60	0.67	9.66	0.70	0.15
Medicare	1.00	0.24	1.61	0.62	1.00
Curative	0.99	0.22	1.54	0.64	0.97
Non‐curative	2.28	0.25	3.74	1.39	0.00
CollaboRATE	0.89	0.24	1.42	0.56	0.64
ER visits	1.54	0.18	2.21	1.08	0.02
Hospital stays	1.40	0.20	2.07	0.95	0.09
CAHPS42	0.83	0.06	0.93	0.74	0.00
Financial difficulty	1.47	0.08	1.71	1.27	0.00
Loneliness	1.70	0.12	2.13	1.35	0.00
Depression	1.07	0.02	1.12	1.03	0.00
Anxiety	1.04	0.02	1.08	1.00	0.04
Self‐efficacy	0.94	0.04	1.01	0.88	0.11
Nausea	1.95	0.11	2.39	1.59	0.00
Constipation	1.17	0.09	1.40	0.98	0.08
Shortness of breath	1.37	0.11	1.71	1.10	0.00
Insomnia	1.92	0.11	2.36	1.56	0.00
CCI^a^	1.09	0.03	1.16	1.02	0.01

^a^ Charlson Comorbidity Index.

### 3.6 High Versus Average QoL Group

Table 4 describes predictors of membership in the high QoL group compared to the average QoL group. Results revealed that college education significantly increased the likelihood of belonging to the high QoL group, by 89%. A higher CollaboRATE and CAHPS42 scores were a significant positive predictor of the high QoL group, while financial difficulties and loneliness had the opposite effect, reducing the likelihood of belonging to the high QoL group by 34% and 38%, respectively. Healthcare utilizations, including ER visits and inpatient stays, reduced the likelihood of belonging to the high QoL group by 31% and 56%, respectively. Depression, anxiety, and comorbid conditions were also associated with 4%–10% decrease in the likelihood of belonging to the high QoL group. Additionally, greater PRO‐CTCAE symptom burden was consistently linked to a lower likelihood of belonging to the high QoL group, with insomnia severity emerging as the strongest negative predictor.

### 3.7 Low Versus Average QoL Group

Table 5 depicts predictors of belonging to the low QoL group compared to the average QoL group. Those receiving non‐curative treatment were 2.28 times more likely to be in the low QoL group. Similarly, those with ER visits were 54% more likely to belong in the low QoL group, though hospital stays were not a significant predictor. In contrast, a higher CAHPS42 score was associated with a reduced likelihood of belonging to the low QoL group. Financial difficulties (47% more likely) and greater loneliness (70% more likely) were both strong predictors of low QoL. Additionally, depression, anxiety, and comorbid conditions were associated with a 4%–9% increase in the likelihood of low QoL. Individuals who experienced greater PRO‐CTCAE symptom burden were also more likely to be in the low QoL group, with nausea and insomnia emerging as the strongest negative predictors.

## 4 Discussion

Using prospectively collected FACT‐G7 scores over a 12‐month period, this study identified three distinct QoL trajectory groups among cancer patients: high, average, and low QoL. The high QoL group consistently demonstrated the highest FACT‐G7 scores across the 12‐month period and showed the greatest increase in scores over time. In contrast, the low QoL group, comprising nearly 20% of the participants, maintained scores below 13 throughout the 12‐month period and exhibited the smallest improvement among the three groups.

The average QoL group was used as a reference group for identifying predictors of the high and low QoL groups, using multinomial logistic regression. Overall, psychosocial factors such as loneliness, financial difficulties, depression, and anxiety significantly increased the likelihood of belonging to lower QoL trajectories. Clinical factors, including comorbid conditions and symptoms of insomnia and nausea, were associated with lower QoL. In contrast, educational attainment and CAHPS42 scores emerged as protective factors and increased the odds of belonging to the high QoL group.

The findings of this study align with the foundational structure of the Wilson and Clearly model, which conceptualizes QoL as a multidimensional construct influenced by the causal pathway of various domains. As hypothesized in our extended conceptual model, QoL trajectories were shaped by an interplay of clinical, psychosocial, sociodemographic, and healthcare system‐related variables. The predicted directions of associations were largely confirmed, with greater symptom burden, psychological distress, and healthcare utilization predicting lower QoL trajectories, and higher education and positive care experiences predicting higher QoL trajectories. These findings not only reinforce our conceptual model but also align with and extend on previous research on QoL in cancer patients.

Specifically, our findings on QoL trajectories align with prior research on breast cancer patients during the first year after the treatment, where three trajectories of low (21%), moderate (45%), and high (34%) QoL were identified [10]. The similarity in trajectory patterns and proportions between our study and the previous studies support the generalizability of these QoL trajectories across different cancer populations. Previous studies have reported that older females with severe economic burden and higher levels of depression are more likely to belong to the low QoL group compared to the average QoL group [11]. Higher educational attainment was found to increase the probability of belonging to the overall high QoL group for patients in their last year of life [13]. Similar sociodemographic and psychological factors were identified as significant predictors of the low QoL group in our study. However, unlike previous findings [11], age and sex were not significant predictors of the QoL trajectories. This difference may be due to the broader age range in our sample, which could have introduced greater variability in life stage and health status, or interactions with other factors.

Regarding healthcare utilization, previous literature found that the low overall QoL group was significantly associated with longer hospital stays, but not with emergency department visits among patients in their last year of life [13]. In contrast, our study found that ER visits were consistently associated with lower QoL group, whereas hospital stays were only significantly associated with a reduced probability of belonging to the high QoL groups compared to the average QoL groups. This discrepancy may reflect the differences in the trajectory and care needs between general care populations and those receiving end‐of‐life care. In terms of clinical factors, prior studies have found that PRO‐CTCAE symptoms such as nausea, shortness of breath, constipation, and insomnia are all negatively associated with QoL in patients with various types of cancer [38]. Our findings align with these results, showing that greater PRO‐CTCAE symptom burden in these domains consistently predicted membership in lower QoL trajectory groups. Lastly, we found that increased comorbidities were consistently associated with lower QoL trajectories, which aligns with previous studies where negative correlations were found between comorbid conditions and QoL, including domains such as global health and physical functioning in breast and prostate cancer patients [39, 40]. While previous studies have largely focused on specific cancer types, our study contributes to the broader literature by examining a more diverse cancer population, with the most prevalent types being breast (31%), lymphoma (11%), leukemia (9%), colorectal (6%), and other gastrointestinal cancers (9%).

Notably, although the direction of associations was consistent across all comparisons, several predictors demonstrated contrast‐specific statistical significance. Specifically, college education, CollaboRATE scores, inpatient stays, and constipation severity only distinguished the high QoL group from the average QoL group, while non‐curative treatment was only associated with membership in the low QoL group compared to the average QoL group. These contrast‐specific patterns suggest that there may be distinct underlying mechanisms across different QoL trajectories. For instance, education and shared‐decision making may help maintain high QoL under moderate symptom burden but may be insufficient to prevent substantial QoL decline when clinical burdens become severe. Similarly, inpatient hospitalization and constipation severity may be most informative in distinguishing individuals with average QoL (relative to high QoL), while treatment intent may primarily signal risk of poor overall QoL.

### 4.1 Study Limitations

Our study findings should be viewed in light of some limitations. Although much larger than prior studies, the study sample was predominantly female (66%), non‐Hispanic (84%), and highly educated (62%). Further, patients were recruited from a single healthcare system and were predominantly privately insured (50%). These characteristics limit the generalizability of our findings to more socio‐economically and racially diverse cancer populations. Specifically, QoL trajectories may differ among men, racial and ethnic minority groups, and individuals with lower education or without private insurance, reflecting potential differences in symptom profiles, psychosocial experiences, and healthcare resources. Future research with more diverse samples is needed to evaluate whether QoL trajectory structures vary across demographic and clinical subgroups. Our study also relied on self‐reported measures, which come with the risk of recall bias and missing data. Future studies may consider the inclusion of objective clinical data. Lastly, time‐varying predictors were not included due to substantial missingness at follow‐up and increased model complexity. Future studies with more complete longitudinal covariate data should examine time‐varying predictors to better understand the temporal associations with QoL trajectories and identify time‐sensitive intervention opportunities.

### 4.2 Clinical and Research Implications

Identifying three distinct QoL trajectories and their corresponding predictors carries several important clinical implications. First and foremost, our findings offer valuable insights into clinical care and resource allocation for the most vulnerable patients. Clinicians can utilize group membership to inform care planning, including decision‐making and follow‐up needs. For example, patients belonging to the consistently low QoL trajectory group may benefit from more intensive, tailored psychosocial or medical interventions, whereas a hands‐off approach or simpler preventive interventions may be sufficient for the average and high QoL trajectory groups. Further, understanding predictors of belonging to a specific trajectory enables earlier intervention by clinicians before QoL deteriorates further.

Our findings also offer additional avenues for intervention development and future clinical trials. Identifying predictors of membership in a specific QoL trajectory can contribute to the development of targeted interventions. For instance, proactive symptom monitoring platforms and psychosocial support interventions (e.g., mental health screening) may help improve or stabilize QoL. Developing sleep‐focused therapy or structured financial navigation programs may be especially beneficial for patients who are likely to belong to the persistent low QoL trajectory. Furthermore, shared‐decision making and communication enhancement programs may be beneficial in helping patients maintain a persistently high QoL trajectory or improve those in the average QoL trajectory. More broadly, identifying these predictors also supports conducting further subgroup analyses in future clinical trials, which may improve intervention targeting across heterogeneous patient populations.

### 4.3 Conclusions

Patient‐reported QoL offers valuable subjective insight into a patient's well‐being and is sensitive to physical, psychological, social, and functional changes over time. Distinguishing unique trajectories of QoL and identifying modifiable risk and protective factors can inform targeted early interventions to improve long‐term QoL outcomes for the most vulnerable patients. This study demonstrates the importance of routine QoL monitoring and highlights specific areas where interventions may be most effective in supporting cancer patients throughout their care journey.

## Author Contributions

**Jiwon Kim:** conceptualization, formal analysis, methodology, visualization, writing – original draft and editing. **Karen Llave:** conceptualization, validation, writing – review and editing. **Maja Kuharic:** conceptualization, formal analysis, methodology, writing – review and editing, project administration, supervision. **Nicola Lancki:** conceptualization, data curation, validation, writing – review and editing. **Kathryn Jackson:** conceptualization, validation, writing – review and editing. **Kimberly A. Webster:** conceptualization, validation, writing – review and editing. **David Cella:** conceptualization, validation, writing – review and editing, funding acquisition, supervision.

## Funding

This work was supported by the National Cancer Institute (UM1CA233035) and the National Center for Advancing Translational Sciences (UL1TR001422).

## Conflicts of Interest

The authors declare no conflicts of interest.

## Supporting information


**Table S1:** Latent Class Descriptives (Mean or %).

## Data Availability

Following publication, the data supporting the findings of this study will be openly accessible in the HealthMeasures Dataverse at https://dataverse.harvard.edu/dataverse/HealthMeasures.
