# Supplementary material for: Identifying Longitudinal Trajectories of Quality of Life and Associated Risk and Protective Factors Among Cancer Patients
Source: Psychooncology. 2026 Apr 6;35(4):e70453. doi: 10.1002/pon.70453 (PMC13051762; doi:10.1002/pon.70453)
Supplement: Supplementary file 1 — Table S1: Latent Class Descriptives (Mean or %). [file PON-35-e70453-s001.docx]

**Supplementary Table.** *Latent Class Descriptives (Mean or %)*

| **Predictor** | **High**  **(n = 1533)** | **Average**  **(n = 1539)** | **Low**  **(n = 747)** |
| --- | --- | --- | --- |
| Age | 62.15 | 60.67 | 57.82 |
| Female | 64% | 65% | 72% |
| College | 95% | 92% | 90% |
| Employed | 47% | 45% | 40% |
| Medicaid | 1% | 3% | 8% |
| Medicare | 43% | 42% | 36% |
| Curative | 17% | 21% | 22% |
| Non-curative | 8% | 12% | 18% |
| CollaboRATE | 46% | 29% | 21% |
| ER visits | 44% | 55% | 64% |
| Hospital stays | 18% | 32% | 42% |
| CAHPS42 | 9.40 | 8.92 | 8.40 |
| Financial difficulty | 1.54 | 2.01 | 2.77 |
| Loneliness | 1.48 | 2.11 | 2.76 |
| Depression | 43.86 | 50.55 | 56.50 |
| Anxiety | 45.76 | 52.81 | 58.92 |
| Self-efficacy | 15.21 | 14.99 | 14.37 |
| Nausea | 1.07 | 1.24 | 1.82 |
| Constipation | 1.31 | 1.60 | 1.98 |
| Shortness of breath | 1.26 | 1.60 | 2.04 |
| Insomnia | 1.62 | 2.22 | 2.87 |
| CCI^†^ | 5.67 | 6.10 | 6.33 |

^†^*Charlson Comorbidity Index*
